# Effectiveness of Flow Diverter Stents in the Treatment of Intracranial Aneurysms: Single-Center 10-Year Results

**DOI:** 10.7759/cureus.74063

**Published:** 2024-11-20

**Authors:** Cemre Ozenbas, Suleyman Men, Ozkan Alatas, Mehmet Akif Kamar

**Affiliations:** 1 Radiology, Tınaztepe University Buca Hospital, Izmir, TUR; 2 Radiology, Dokuz Eylul University Research and Application Hospital, Izmir, TUR; 3 Radiology, Izmir State Hospital, Izmir, TUR; 4 Radiology, Kırsehir State Hospital, Kırsehir, TUR

**Keywords:** endovascular treatment, flow diverter stent, interventional radiology, intracranial aneurysm, radiology

## Abstract

Purpose

This study's objective was to show how effectively flow-diverting stents work when treating cerebral aneurysms. The assessment aimed to determine efficaciousness related to aneurysm type, size, and location as well as early- and late-term results.

Material and method

The study included 153 aneurysms from 122 patients treated in interventional radiology unit with flow diverter stents between July 2010 and June 2020. Apart from demographic data, size, type, and location of aneurysms; technical success, peri, post-procedural, and late complications; aneurysm occlusion rates, morbidity and mortality rates were extracted from patient records.

Results

This study enrolled 90 (73.8%) females and 32 (26.2%) males; average age of the patients was 52.8±12.9. The average follow-up period was 33.4±9.9 months. The most common type of aneurysm was saccular (133; 86.9%), and the most common location was the supraclinoid segment with 67 (43.8%). Complete occlusion was observed in 115 of 140 aneurysms from 110 patients during follow-up. No statistically significant difference was detected between the sizes and types of aneurysms and complete occlusion rates (p=0.096, p=0.583). The total occlusion rate was found to be significantly higher in internal carotid artery (ICA) supraclinoid segment aneurysms compared to other locations (p = 0.006). During the follow-up period, flow diverter stent-related permanent morbidity was observed in four (3.6%) patients and mortality was observed in five (4.5%) patients.

Conclusion

Flow diverter stents can be successfully applied to many aneurysm types, from small aneurysms to large and giant aneurysms, and have high aneurysm occlusion rates with low morbidity and mortality. By the aspects of the current study the total occlusion rate was found to be significantly higher in ICA supraclinoid segment aneurysms compared to other localizations.

## Introduction

The prevalence of intracranial aneurysm has been reported between 0.4% and 8% in different large-scale studies [1]. Although they are commonly asymptomatic, rupture may result in subarachnoid hemorrhage which is related to devastating complications. Only 25% of the sufferers of subarachnoid hemorrhage gain previous normal daily psychological and neurological state [2]. The recent endovascular treatment modalities are known as coiling, stent-assisted coiling and flow diverter stents. Endovascular treatment options are advantageous in avoiding craniotomy, reducing hospital stay and less postoperative complications [3].

Flow diverter stents have come to the fore in the treatment of intracranial aneurysms in recent years. Flow diverter stents direct the flow through a bridge created at the neck of the aneurysm, and the slowed blood flow within the aneurysm sac causes stasis, inflammatory response, and thrombosis. In addition, endothelialization developing in the main artery wall at the neck of the aneurysm creates reconstruction in the wall, causing the aneurysm to remain out of circulation [4].

This study is aimed to investigate the effectiveness of flow diverter stents used in the treatment of intracranial aneurysms. Also, their relationship with aneurysm size, type and location.

## Materials and methods

Patient and aneurysm characteristics

The study was conducted after the approval of Dokuz Eylul University Non-invasive Research Ethics Committee (approval 5791-GOA) and in the light of the Helsinki Declaration. For this retrospective study patients whose intracranial aneurysms were treated with flow diverter stents between July 2010 and June 2020 were enrolled. Any previous coil or clip treatments were assessed as exclusion criteria. Age and gender information of the patients and the number, size, type and location of aneurysms were recorded. Written informed consent form was obtained from all patients before the procedure.

At the initial stage of the study period we treated only giant and large cerebral aneurysms with flow diverters. Later we started to treat all size sidewall aneurysms and some selected bifurcation aneurysms that are difficult to treat by other endovascular methods.

For the ruptured saccular aneurysms we prefer balloon-assisted coiling. If we have to use stent-assisted coiling in very wide-necked ruptured side wall aneurysms we prefer to use flow diverters in addition to coiling. We also treat the ruptured blister aneurysms with flow diverters. We load 30 mg prasugrel of 180 mg ticagrelor three hours before the procedure in cases of ruptured aneurysm.

Pre-procedure

Before the procedure, antiplatelet loading was performed. In the initial stages, dual antiplatelets (clopidogrel and acetyl salicylic acid (ASA)) were given, while prasugrel was used as a single drug in the later stages. 450 mg clopidogrel and 300 mg ASA were given the night before the procedure, which will be continued with ASA (100 mg/day) and clopidogrel (75 mg/day). A resistance test was performed with the multiplate system on the morning of the procedure. The drug was considered effective at values ​​if the area under curve for aspirin was below 330 U for ASA and area under curve for adenosine diphosphate (ADP) was below 40 U for clopidogrel. A 30 mg oral prasugrel loading was given two hours before the procedure, with the maintenance dose of 10 mg/day continuing with prasugrel. The resistance test was performed again measuring the area under curve for ADP with the multiplate system. If the the patient is resistant ​​ticagrelor 180 mg was given and the test was repeated three hours later.

Endovascular procedure

All procedures were performed under general anesthesia by an experienced neuro-interventionalist (SM) in a biplane neuro-angiography unit. After accessing the femoral artery under ultrasonography guidance, 7F 80cm Arrow-Flex (Teleflex, Inc., Morrisville, NC, USA) or 7F 90 cm Ebsylar introducer sheath (Optimed, Ettlingen, Germany) was placed into the descending aorta. After the target artery was catheterized with the diagnostic catheter advanced through the guiding catheter, the 7F sheath was advanced to the target artery. Then, the Fargo (Balt, Montmorency, France) distal access catheter was advanced to the distal cervical internal carotid artery (ICA) for anterior circulation aneurysms, and to distal cervical vertebral artery for posterior circulation aneurysms. Then diagnostic angiography including 3D rotational angiography was obtained and the appropriate working view is determined. Vasco+21 microcatheter (Balt) was used for delivery of Silk (Balt) stents and Marksman 27 microcatheter (Penumbra, Alameda, CA, USA) for pipeline endovascular device (PED, Covidien/Medtronic, Irvine, CA, USA). Generally the stents were placed by pushing and pulling maneuvers of the microcatheter. Segmental malalignment that occurred during deployment was corrected with balloon manipulations. In cases of persistent malalignment at the ends of the flow diverter stent additional non-flow diverter metal stents including Leo stent (Balt) or channel balloon-expandable stent were deployed to ensure an undisturbed flow in the stented artery. In patients with carotid artery aneurysms, the stent was usually chosen long enough to extend to the middle cerebral artery (MCA) M1 segment for stability. In patients without an anterior communicating artery or with both anterior cerebral arteries filling from the ipsilateral carotid circulation the MCA M1 segment was not covered. If the neck of the aneurysm was too wide especially in giant aneurysms a long Leo stent was placed first to provide a safe frame for the Silk flow diverter. Then the Silk stent was placed coaxially.

During the procedure, all patients received a 5000 IU heparin bolus. At the end of the procedure, possible hemorrhagic complications were ruled out with flat panel CT. Furthermore, a regular CT head was obtained before and after the procedure in all patients. After the procedure, the heparin effect was left to resolve on its own.

Angiographic images during the procedure were evaluated by experienced practitioners SM and CO in terms of technical success in stent placement, stent type and developing complications. In cases of disagreement, the decision was taken jointly.

Post-procedure follow-up

Patients receiving dual antiplatelet treatment took ASA 100 mg/day and clopidogrel 75 mg/day. Prasugrel 10 mg/day or ticagrelor 90 mg twice a day was given as a single drug. In both methods, ASA 100 mg/day use was continued for life after six months. Prophylactic 16 mg dexamethasone was started on patients with giant aneurysms after the procedure, and the dose was tapered and stopped within three weeks.

After treatment, patients were followed up with angiography at six months, and annually thereafter. At the sixth-month follow-up a catheter angiography was done in all patients. If the aneurysm totally disappeared angiographically and no other angiographic abnormality existed, further follow-up imaging was done with magnetic resonance angiography (MRA) and magnetic resonance imaging (MRI). Otherwise follow-up exams were done as catheter angiography at annual intervals. In follow-up examinations, the degree of aneurysm occlusion was classified according to the O'Kelly-Marotta (OKM) Scale as complete filling (Grade A), filling in the entry segment (Grade B), filling in the neck segment (Grade C), and complete occlusion (Grade D) [5]. Complications in follow-up examinations were classified as hemorrhagic, thromboembolic and mass effect.

Statistics

IBM SPSS 21.0 program (IBM Corp., Armonk, NY, USA) was used for data analysis. Descriptive statistics and frequency distributions were evaluated. Numerical variables are given as mean and standard deviation and median (minimum-maximum). After testing normal distribution Pearson Chi-Square Test was used to show whether there was a statistically significant difference between the frequencies. For a statistically significant difference, a p value of less than 0.05 was accepted.

## Results

Characteristics of patients and aneurysms

One hundred fifty-three aneurysms from 122 patients (90 women; age range, 22-84 years; mean age, 52.8±12.9) were included in the study. Forty-one of the aneurysms were small (<5 mm), 55 were medium (5-10 mm), 42 were large (10-25 mm), and 15 were giant (>25 mm). When aneurysms were evaluated according to their location, 139 (90.9%) were in the anterior circulation and 14 (9.1%) were in the posterior circulation. The most common location was the ICA supraclinoid segment with 67 aneurysms (43.8%).

Eleven aneurysms in 11 patients were ruptured while the rest were unruptured (142 aneurysms in 111 patients). Seven aneurysms were ruptured blister aneurysms and treated with Silk flow diverters. Three saccular ruptured aneurysms were first treated with balloon-assisted coiling and then the very wide neck was secured with a Silk flow diverter. One ruptured aneurysm was actually a pseudoaneurysm with an accompanying carotid cavernous fistula created by a craniofacial trauma five days ago. The pseudoaneurysm was coiled with balloon assistance and the parent artery was reconstructed with a Silk flow diverter at the end. Characteristics of patients and aneurysms are shown in Table 1.

**Table 1:** Demographic information of the patients and radiological features of the aneurysms MCA: Middle cerebral artery, ICA: Internal carotid artery, ACA: Anterior cerebral artery, Acom A: Anterior communicationg artery, PCA: posterior cerebral artery, Pcom A: posterior communicationg artery

Gender	N (%)
Female	90(73.8%)
Male	32(26.2%)
Aneurysm Size	
Small	41(26.8%)
Medium	55(35.9%)
Large	42(27.5%)
Giant	15(%9.8)
Aneurysm Type	
Saccular	133(86.9%)
Dissecting	7(4.6%)
Blister	7(4.6%)
Fusiform	6(4%)
Aneurysm Localization	
ICA Supraclinoid	67(43.8%)
ICA Cavernous	21(13.7%)
MCA	14(9.2%)
Vertebrobasilar	14(9.2%)
ICA Paraclinoid	12(7.8%)
ACA	9(5.9%)
Acom A	6(3.9%)
ICA Bifurcation	5(3.3%)
Fetal PCA	3(2%)
Pcom A	2(2%)

Intraoperative and early results

**Figure 1:**
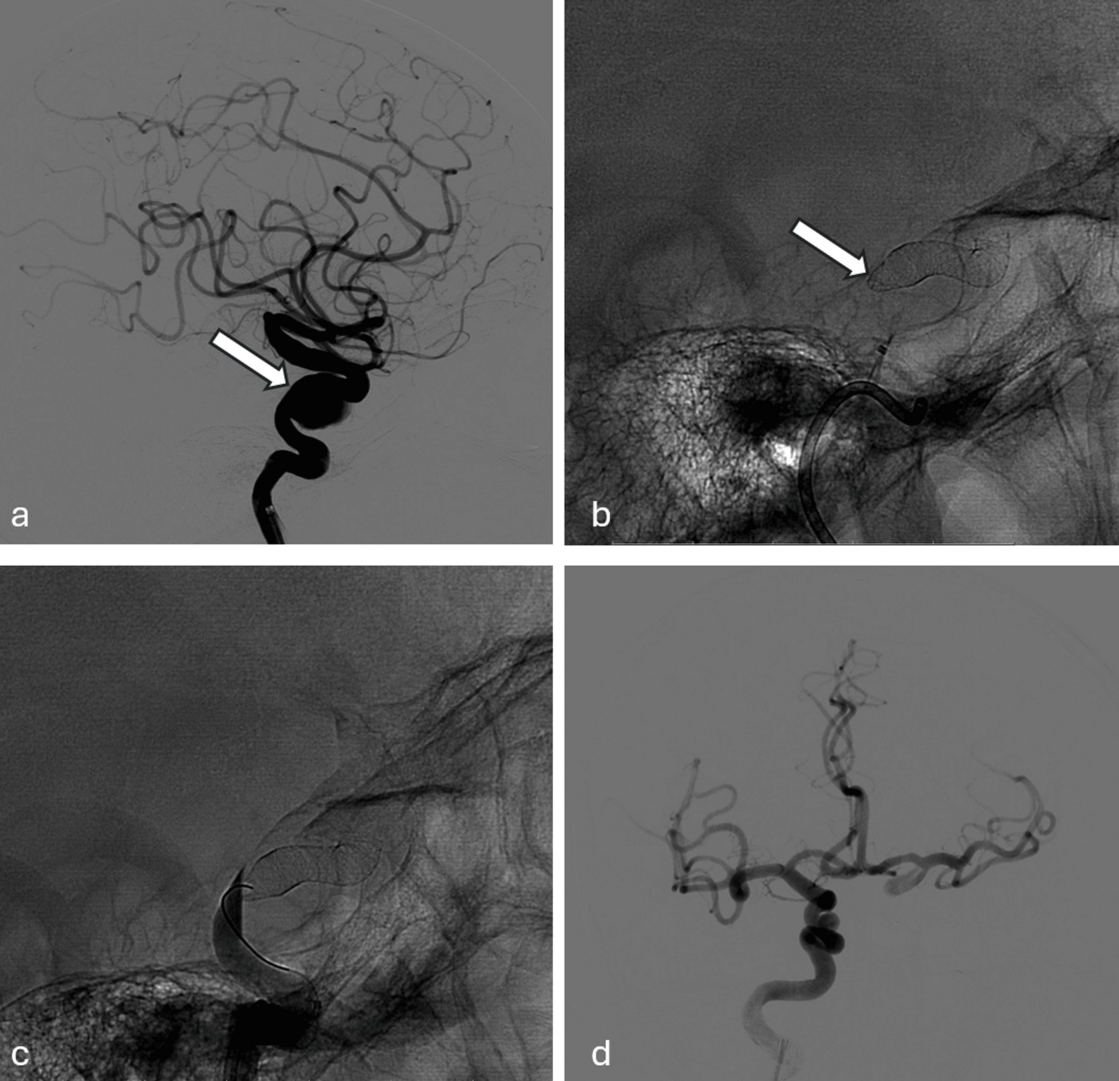
a: Aneurysm (white arrow) in the internal carotid artery (ICA) cavernous segment in the subtraction angiography images. b: Collapse of the proximal part of the stent in unsubtraction pre-injection images (white arrow). c: Unsubtracted angiography image shows that the stent is thrombosed. d: Vascular structures showing complete and simultaneous filling on the aneurysm side via the communicating artery in the subtracted angiography images of the contralateral injection.

The stent type used in the treatment of the aneurysm was Silk in 139 (90.8%), pipeline in 13 (8.4%), and Silk and pipeline flow diverter stents together in one (0.8%) patient. For six (2.6%) aneurysms, coiling was used in addition to flow diverter stents. Of these coiled aneurysms four were ruptured (mentioned above) and two were giant aneurysms. Coiling was used to decrease the clot burden. In two patients the proximal end of the device (pipeline in one and Silk in one) was collapsed and deformed, which ended with occlusion of the stented carotid artery together with the aneurysm. These two patients well tolerated the occlusion thanks to efficiently working communicating arteries (Figure 1). In one patient the proximal end of the stent was angled into the aneurysm sac without any flow disturbance. This stent was not further manipulated and left as it was. In the remaining patients all devices were placed into the desired positions. In nine patients nine Leo stents were adjunctively used to construct an initial bridge or correct the proximal or distal malposition of the flow diverter. In one patient a channel balloon expandable stent was telescopically opened to restore luminal patency in the proximal end of an under-expanded Silk stent.

In seven patients the flow diverter stent was totally thrombosed after uneventful deployment during the procedure. These stents were again totally recanalized with intravenous tirofiban infusion and balloon dilatation within the stent (to create a hole for exposure of the drug) within approximately an hour. Six of these seven patients woke up without any neurologic deficit. In only one of these patients multiple foci of small infarcts developed which resulted in minor stroke. The patient was independent when she came to the six-month follow-up imaging.

In one patient the subcallosal artery was occluded and the patient developed antegrade amnesia. She improved during the follow-up and was independent at six months. Five other patients woke up with minor stroke although the procedure was technically uneventful. These patients also improved and were not disabled during the follow-up. Modified Rankin Scores (mRS) were determined as 0 and 1.

In two patients treated giant aneurysms ruptured two days and five days after the procedure, which resulted in death. One patient developed intracerebral hemorrhage in the hemisphere contralateral to the stent on the fourth day. The hemorrhage resolved within months with the patient free of neurologic deficit in the follow-up.

One patient with subarachnoid hemorrhage that resulted from a blister aneurysm of the basilar artery was treated with flow diverter. Three weeks later the patient died of central nervous system infection that developed after external ventricular drainage for acute hydrocephalus.

**Figure 2:**
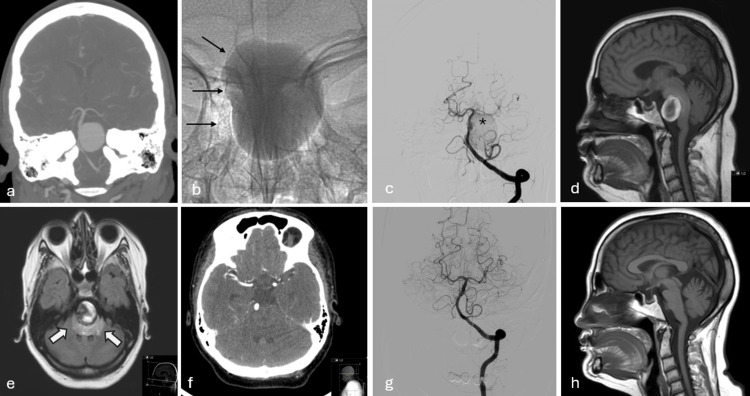
a: Coronal reformatted computed tomography angiogram shows a giant mid basilar aneurysm. b: The unsubracted image shows the Silk stent (arrows) deployed against the aneurysm neck. Note the contrast medium from the previous injection is stagnated within the aneurysm. c: Post stent vertebrobasilar angiography shows very faint opacification of the giant aneurysm (*) because of the stagnation within the aneurysm. d: The patient presented with gait disturbance and imbalance on the third month. Sagittal T1-weighted image reveals distortion of the brain stem by the thrombosed aneurysm. e: Axial fluid-attenuated inversion recovery (FLAIR) image shows severe vasogenic edema (arrows) surrounding the clotted aneurysm. f: CT angiography confirms total thrombosis of the aneurysm. g: Nine-month follow-up angiography shows no filling of the aneurysm. h: Six-year follow-up. Sagittal T1 image almost total disappearance of the aneurysm and recovery of the brain stem anatomy. A very small remnant of the sac is seen anterior to pons.

Three patients with giant aneurysms experienced neurologic deficit associated with mass effect. Two of the developed hydrocephalus resulting from aqueducts cerebri compression in one and foramen of Monro in the other. Both patients underwent ventriculoperitoneal shunting. The symptoms resolved in both patients, however one of the patients subsequently died of shunt infection. The mass effect resulting from a giant basilar artery aneurysm in the third patient totally resolved with prolonged corticosteroid treatment (Figure 2). 

Excluding the clinically uneventful recanalization of acute stent thrombosis in six patients and clinically tolerated carotid artery occlusion in two patients, thromboembolic complications associated with neurologic deficit occurred in seven patients, death in four patients, and neurologic deficit associated with mass effect of the aneurysm in three patients in the hospitalization period.

Follow-up results

Because eight patients with nine aneurysms were lost to follow-up and four patients with four aneurysms died in the peri-procedural period, the evaluation of the follow-up findings was made on 140 aneurysms. During the follow-up, 100 aneurysms (71.4%) were occluded in the first nine months, seven aneurysms (5%) were occluded between 10-23 months, and eight aneurysms (5.7%) were occluded in the 24th month or later. During the entire follow-up period, which averaged 33.4±9.9 months, 115 of the aneurysms (82.1%) remained completely out of circulation (OKM Grade D). Fifteen aneurysms (10.7%) decreased in size, but partial filling continued (OKM Grade B). In 10 of them (7.1%), no difference was detected according to pre-treatment examinations (OKM Grade A). The occlusion status of the aneurysms and the periods of occlusion shown in Table 2.

**Table 2:** Occlusion status of aneurysms and the periods of occlusion OKM: O'Kelly-Marotta Scale

Occlusion Status	N (%)
Total occlusion (OKM Grade D)	115(82.1%)
Subtotal occlusion (OKM Grade B)	15(10.7%)
No occlusion (OKM Grade A)	10(7.1%)
Periods of occlusion	
0-9 Months	100(71.4%)
10-23 Months	7(5%)
24 Months and above	8(5.7%)

The characteristics of aneurysms before intervention were compared with total occlusion rates in follow-up examinations, and when comparing complete occlusion frequencies across different aneurysm size groups, the p-value was found to be 0.096 (p > 0.05), indicating no statistically significant difference among size categories. Similarly, the relationship between aneurysm types and post-intervention total occlusion rates yielded a p-value of 0.583 (p > 0.05), also showing no statistically significant difference. However, a statistically significant difference was observed when comparing the relationship between aneurysm localization and post-intervention total occlusion rates, with a p-value of 0.002 (p < 0.05). Total occlusion was achieved in 92.1% of aneurysms located in the supraclinoid segment of the ICA, whereas this rate was 74% for aneurysms in other locations. The chi-square test confirmed this difference, with a p-value of 0.006 (p < 0.05).

**Figure 3:**
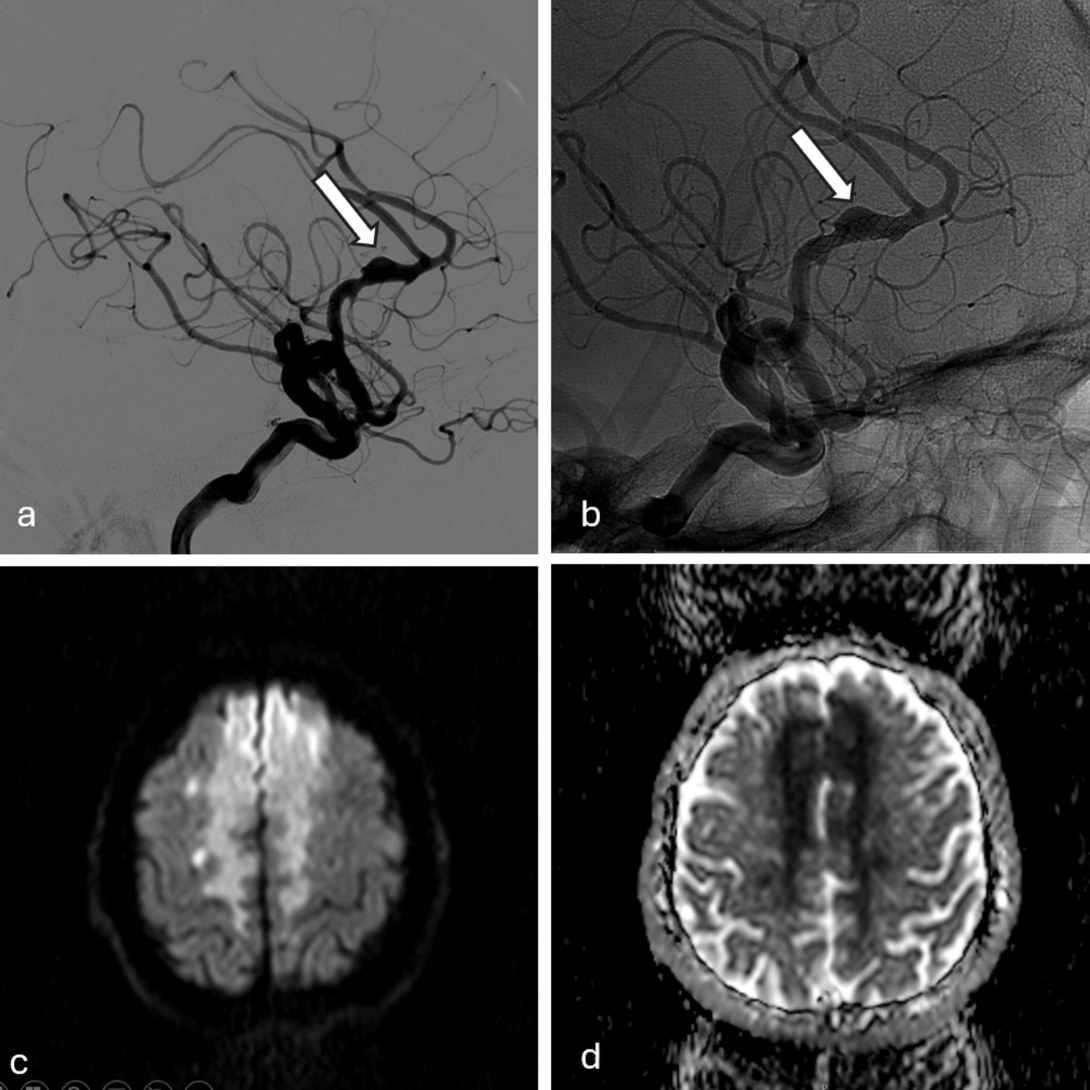
a: Fusiform aneurysm (white arrow) in the pericallosal artery in the subtracted angiography images. b: Silk stent and fusiform aneurysm (white arrow) in unsubstracted angiography images. c and d: In the diffusion-weighted imaging (DWI) trace and apparent diffusion coefficient (ADC) images an acute infarction in the anterior cerebral artery (ACA) territory.

Delayed ischemic complications were seen in 11 patients. A supraclinoid carotid stent thrombosed following scheduled cease of clopidogrel (while ASA was continued) after six months in one patient and another azygos pericallosal stent thrombosed following cease of clopidogrel by the patient himself after two months (Figure 3). The occlusion resulted in massive infarction leading to disabling stroke in these two patients. In one patient the cavernous carotid stent together with the aneurysm was totally occluded at the sixth month angiography while the patient was asymptomatic. Three patients with basilar artery stents developed perforating artery infarction six, eight and 23 months following stenting. Two patients with middle cerebral artery and two patients with anterior cerebral artery stents developed small perforator infarcts six months, 12 months, 13 months and two years after stenting. Six patients with carotid stents traversing the ophthalmic artery origin experienced retinal transient ischemic attacks after scheduled stopping of clopidogrel or prasugrel. Clopidogrel was restarted in these patients.

Delayed hemorrhage occurred in one patient. This was a giant M1 segment aneurysm treated with stent (Leo+Silk) assisted coil embolization. Although the aneurysm was totally occluded on follow-up angiography, in the fourth year of follow-up the patient had severe subarachnoid and intraventricular hemorrhage that led to death within two days. 

Intimal hyperplasia causing in-stent stenosis was seen in eight patients. The degree of stenosis was 75% in two patients and mild in the other patients. No intervention was done in these patients because they were not symptomatic. In follow-up examinations, the stenosis resolved at 12 and 24 months.

Overall 14 patients experienced minor ischemic stroke that ended up with an mRS 0 or 1, two patients had disabling ischemic stroke, one patient had hemorrhagic stroke with a final mRS 0, and five patients died as a complication of early or late hemorrhage, mass effect, and subarachnoid hemorrhage. 

## Discussion

Endovascular treatment options for aneurysms have multiple advantages over surgical options, therefore they have been more commonly used in appropriate cases. By the results of the current study the complete occlusion rate was found to be 84.5% in aneurysms of all sizes, types and locations. Also, evaluation of the long-term results reveals that more successful results were obtained in ICA supraclinoid segment aneurysms compared to other locations. Aneurysm occlusion and complication rates, morbidity and mortality rates were consistent with the current literature.

While treatment with flow diverter stents seems to be beneficial it is not exempt from complications. Thromboembolic complications are the most common periprocedural complications and related to morbidity and mortality. Hyperacute occlusion of the stent may result from malapposition of the stent [6]. We have corrected the inappropriately deployed stent ends by balloon angioplasty or by deploying additional adjunctive stents in nine patients. However correction was not possible in two patients, which resulted in occlusion. Fortunately the patients tolerated the occlusion. These two patients were treated at the initial stage of the study when our experience was limited. Acute intrastent thrombosis may occur in a widely open well-apposed stent as seen in seven patients in this study. The rate of acute thrombosis of flow diverter stents has been reported to be between 2% to 7.5% [7-11]. Although all of our patients were tested for antiplatelet sensitivity, thrombosis did occur in some of them. One explanatory point may be that platelet reactivity to antiplatelet drugs is not the same in every individual and there is no consensus on the best method to measure the preprocedural antiplatelet response [12,13]. Hohenststt et al. reported 12 cases of acute intrastent thrombosis out of 166 procedures and advised an active surveillance of 30 minutes after implantation of the flow diverter for early detection and prompt treatment [14].

Delayed ischemic events were also seen in the present study. One patient experienced azygos anterior cerebral artery flow diverter stent thrombosis after unplanned discontinuation of clopidogrel at two months and another experienced carotid flow diverter stent thrombosis after planned discontinuation of clopidogrel at six months. Both patients were still on ASA 100 mg/day. Both patients developed disabling stroke. The first case can be explained by the patient’s uncompliance; it is hard to explain the second. Delayed stroke associated with in-stent thrombosis up to 23 months while on aspirin has been reported before [15-17]. There is not enough evidence to define the safe duration of dual antiplatelet treatment. Another cause of late ischemic complications is perforator occlusion. Perforators particularly in the posterior circulation are at high risk for infarction in flow diverter treatments [18]. Neointimal overgrowth narrowing of the perforator orifice could be the potential cause. In our study the overall frequency of thromboembolic complications that resulted in minor neurologic deficit was 11.4% (14 patients) and frequency of disabling stroke was 1.6% (two patients). The frequency of ischemic stroke was determined by Fujii et al. which including large and giant aneurysms, was 3.6%; while in the study of Raychev et al. which was evaluating patients treated with pipeline stents was 6%; and Wang et al. found it to be 11% in their meta-analysis where they included posterior system aneurysms [19-21].

The propensity of very large and giant aneurysms to rupture is well-known and may be associated with intraaneurysmal thrombus formation [22,23]. Kulcsar et al. in a post-mortem study have observed the following common features of aneurysms that ruptured after flow diverter treatment: 1) Large and giant aneurysms potentially able to contain large rapidly accumulated thrombi, 2) Symptomatic aneurysms suggesting recent growth and wall instability, 3) Saccular aneurysms with dome height to neck ratio>1.6, 4) Morphological characteristics predisposing to an inertia-driven flow.

Delayed aneurysm rupture is a rare complication that can result in mortality. In our study two giant aneurysms ruptured in the periprocedural period and one giant aneurysm ruptured 4.5 years after treatment. Kulcsar et al. in their study of 1421 aneurysms, detected delayed aneurysm rupture in 14 patients (1%). All aneurysms except one were shown to be large or giant aneurysms. In four of these patients, rupture occurred more than three months after the procedure, and the latest rupture occurred 300 days after the treatment. In their study, additional coiling of the stent was not used in any of the patients with rupture. Rouchard et al. in their literature review showed that 50% of the reported late aneurysm ruptures were seen in giant aneurysms and that coiling was used in addition to stents in 20% of them. For this reason, they suggested that coil use may not always be preventive in delayed aneurysm rupture [24]. In our study, a late aneurysm rupture occurred 37 months after the procedure in one patient (0.9%). In this aneurysm, a Silk stent was placed along with coiling for the ICA terminal segment aneurysm and the aneurysm seemed radiologically totally cured during follow-up. This period (37 months) is one of the latest aneurysm ruptures described in the literature to the best of our knowledge. This particular case of ours points to the still uncovered properties of giant aneurysms.

Delayed intraparenchymal hemorrhage is another poorly understood complication of flow diverter treatment. In a metanalysis by Rouchoud et al., its frequency was reported to be 2-3%. While 80% occurred in the ipsilateral vascular territory, the rest was seen in different vascular territories. Various mechanisms like hemodynamic change after flow diversion, hemorrhagic transformation of subtle periprocedural infarcts, and oversensitivity to dual antiplatelet treatment were among the proposed mechanisms. In our study one patient developed intraparenchymal hemorrhage in the contralateral middle cerebral artery territory four days after flow diverter treatment of a giant cavernous carotid artery aneurysm. A thorough radiologic examination of the patient did not provide any evidence to explain the hemorrhage.

A rare complication seen especially in large and giant aneurysms is mass effect and perianeurysmal edema. Although the exact mechanism is not known, it is speculated that the increase in the size of the rapidly thrombosing aneurysm, endothelial damage triggered by thrombus, and inflammation caused by stress signals may play a leading role. Berge et al. in their study of patients with clinical worsening after flow-diverter stent placement, found a correlation between vasogenic edema found in MRI examination and clinical findings. In the same study, it was shown that all patients with this finding had aneurysms embedded in the parenchyma without intervening CSF and that the aneurysms were large in size [25]. In the current study, mass effect and perianeurysmal edema were observed in three patients (2.7%). Similar to the literature, all three aneurysms were giant aneurysms with large and close contact areas with the parenchyma.

Although the development of in-stent stenosis is a well-known process in coronary artery stents, its mechanism in flow diverter stents is not fully understood. Different phases that may contribute to the development of in-stent stenosis in various animal models were defined by Schwartz et al. These factors were thrombus formation and inflammation in the early phase, endothelialization and granulation tissue in the intermediate phase, and low muscle cells and matrix formation in the late phase [26]. John et al. found intimal hyperplasia in 41% of the 51 patients they treated with pipeline stents. Of these, four were classified as mild, one as moderate in-stent stenosis, and the rest as minimal intimal hyperplasia. In their study, the total in-stent stenosis frequency was shown to be 9.8% [27]. In the current study, intimal hyperplasia causing in-stent stenosis was observed in eight patients. Although it caused serious stenosis in two patients, no additional intervention was performed because no significant delay in flow was detected and no symptoms were observed. More comprehensive studies are needed regarding the factors that may cause the development of intimal hyperplasia, the complications it may cause, and the approach to patients.

One of the most important indicators of success in flow diverter stent treatment is the rate of aneurysms remaining out of circulation in follow-up examinations. Brinjikji et al. in a meta-analysis that included 29 studies found that the total occlusion rate varied between 55% and 95%, with an average of 75%. In the study showing that aneurysm size has no effect on treatment success, it was shown that endovascular treatment, which is claimed to be more effective in small-sized aneurysms, also provides very high occlusion rates in large and giant aneurysms. Zhou et al. in a large meta-analysis including 59 studies found the total aneurysm occlusion rate to be 82.5%. In the same study, occlusion rates were found to be 88.7% in blister aneurysms, 80% in small aneurysms, 74% in large aneurysms, and 76% in giant aneurysms, but no statistically significant difference was found between them. In our study, the complete occlusion rate was found to be 82.1% in aneurysms of all sizes, types and locations. No statistically significant difference was found between aneurysm size (p=0.096), types (p=0.583) and complete occlusion rates. The findings are parallel to the rates in the literature. A statistically significant difference was found between aneurysm localization and complete occlusion rates (p=0.002). When evaluating the localization relationship, statistical evaluation was compared as large groups and the remaining aneurysms due to insufficient numbers in most of the groups. The complete occlusion rate, which was 92.1% in ICA supraclinoid segment aneurysms, was found to be 74% in all remaining aneurysms. Many factors affect aneurysm occlusion rates, such as wide neck, difficult and angled main artery structure, and branch rise within the aneurysm. It may be possible to predict success rates before the procedure with comprehensive studies on the identified factors.

The best indicator of the reliability of flow-diverting stents is the morbidity and mortality rates. In Zhu et al.'s meta-analysis conducted on 2263 patients, permanent morbidity and mortality rates were found to be 6% and 4.3% [28]. Kallmes et al. in a multicenter study evaluating the five-year follow-up period of 793 patients treated with pipeline stents found the total permanent morbidity and mortality rate to be 7.4% and 3.8%. In the same study, the group with the highest mortality was found to be posterior system aneurysms (10.9%) [29]. In our study, the permanent morbidity rate was calculated as 3.6% and the mortality rate was calculated as 4.5%. Three out of five patients with mortality are posterior system aneurysms. Similar to the literature, mortality rates were found to be higher in posterior system aneurysms in our study. It was thought that this situation may be due to the anatomical and access difficulties in posterior system aneurysms and the multiplicity of perforating arteries.

The main limitation of our study is its retrospective nature. The limitations of the study include the diversity in aneurysm localizations, the use of different stents, and the use of two different antiaggregant regimens. Data loss occurred due to patients whose follow-up examinations could not be performed after the procedure. Follow-up examinations are not homogeneous, as 16 patients were followed up with MRI and CT angiography.

## Conclusions

In our study, flow diverter stents used in aneurysm treatment were found to be a treatment method that provides effective treatment in line with the literature and has low morbidity and mortality. For evaluation of the reliability and effectiveness of flow diverter stents, more comprehensive studies with long-term follow-up are needed to better understand the complications they may cause.
